# Effects of Mint Oils on the Human Oral Microbiome: A Pilot Study

**DOI:** 10.3390/microorganisms12081538

**Published:** 2024-07-27

**Authors:** Samar M. Abdelrahman, Manar El Samak, Lamis M. F. El-Baz, Amro M. S. Hanora, Prabodh Satyal, Noura S. Dosoky

**Affiliations:** 1 Department of Botany and Microbiology, Faculty of Science, Suez University, Suez 43518, Egypt; 2 Department of Microbiology & Immunology, College of Pharmacy, Suez Canal University, Ismailia 43221, Egypt; manarelsamak@yahoo.com (M.E.S.); a.hanora@pharm.suez.edu.eg (A.M.S.H.); 3 Department of Zoology, Faculty of Science, Suez University, Suez 43533, Egypt; lamis.albaz@suezuniv.edu.eg; 4 dōTERRA International, Pleasant Grove, UT 84062, USA

**Keywords:** baseline study, halitosis, oral microbiome, periodontal diseases, *Mentha piperita* EO, *Mentha canadensis* EO, *Mentha citrata* EO, *Mentha spicata* EO

## Abstract

The oral microbiome is a diverse and complex ecosystem essential for maintaining oral and systemic health. Our study is the first to define the oral microbial community in Egyptian young adults and investigate the effects of natural antimicrobials on the oral microbiome. SuperMint (SM) is a proprietary blend of peppermint, Japanese mint, bergamot mint, and spearmint essential oils encapsulated in a tiny soft beadlet. This work aimed to evaluate the effects of SM beadlets on the oral microbiome. This study recruited twenty healthy participants. A baseline investigation of the oral microbiome of the selected participants was performed by collecting saliva and swab samples before treatment. Treatment included chewing four SM beadlets twice a day for 7 days, and then, post-administration saliva and swab samples were collected at the end of treatment. The oral microbiome samples were analyzed by the high-throughput amplicon sequencing of 16S rRNA gene fragments, and the community composition was determined. The results showed that the abundance of some microbial genera and families decreased after using SM, including *Prevotella*, *Streptococcus*, *Neisseria*, and *Haemophilus*. However, some genera showed inconsistent patterns. We also found that the subject’s gender and SM usage were significantly associated with diverse microbial composition. The results suggest that SM treatment decreased the abundance of several bacteria associated with halitosis and periodontal diseases, such as *Actinomyces* and *Streptococcus*. Furthermore, *Corynebacterium* species increased and *Streptococcus* decreased after SM usage. More research is needed to fully understand the antimicrobial effects of mint oils and their potential applications in maintaining good oral health.

## 1. Introduction

The human body is colonized by a vast number of microbial populations known as the human microbiome [1,2]. Trillions of bacteria, viruses, fungi, and other microorganisms (about 100 trillion cells) colonize various locations in the body, such as the oral cavity, nasal cavity, gut, reproductive tract, and skin [1,3]. The oral microbiome is a diverse and complex ecosystem consisting of over 1000 species of bacteria, archaea, viruses, fungi, and protozoa. These microorganisms are essential to maintaining good oral health by helping with nutrient digestion and absorption, preventing harmful microorganisms from taking hold, and boosting the immune system [4]. Several factors can affect the oral microbiome, including diet, antibiotic use, stress, oral hygiene practices, genetics, diseases like diabetes, and the environment [5]. One question in current studies is determining whether the contents of toothpaste could be responsible for the alteration of oral commensal–eubiotic flora, favoring the development and accumulation of harmful bacteria. A diet high in sugars can result in tooth decay and gum disease. On the other hand, a fiber- and probiotic-rich diet can encourage the growth of beneficial bacteria that enhance oral health [6]. Poor oral hygiene practices like infrequent brushing and flossing can lead to the accumulation of harmful bacteria and plaque, which can cause dental diseases. Poor oral hygiene is also responsible for gingiva–periodontal pathologies. Moreover, imbalances in the oral microbiome community can have systemic effects on the body [7]. It is important to mention that oral dysbiosis, which is the disruption or imbalance of the normal microbial community, can be caused by poor oral hygiene, medications, smoking, alcohol consumption, and stress. A strong correlation has been reported between periodontal pathogens and an increased risk of respiratory infections, cardiovascular disease, diabetes, and some cancers [8,9,10]. Understanding the dynamic nature of the oral microbiome is essential to developing successful preventive and therapeutic strategies to maintain good oral and systemic health [5].

Several methods are used to study the oral microbiome, including 16S rRNA gene sequencing, metagenomics, culturing bacteria from oral samples, microscopy, and functional assays [1]. One of the most common methods is 16S rRNA gene sequencing, which involves analyzing the DNA of bacterial species in oral samples [11]. This method can identify different bacterial groups by targeting a conserved region of the bacterial genome and provide information on their abundance and diversity. This method involves extracting DNA from oral samples and amplifying the 16S rRNA gene using polymerase chain reaction (PCR). The amplified DNA is then sequenced and analyzed using bioinformatics tools to identify different bacterial groups [11,12]. It is worth mentioning that 16S sequencing also detects dead or inactive bacteria, which can affect the interpretation of results. Another method is metagenomics, which involves the comprehensive sequencing of all the DNA in a sample, including bacterial, viral, and fungal DNA. This method involves extracting DNA from oral samples, sequencing the DNA using high-throughput sequencing technologies, and analyzing the data using bioinformatics tools to identify different microbial groups and determine their functions [13].

SuperMint (SM) is a proprietary blend of peppermint (*Mentha piperita* L.), Japanese mint (*M. arvensis* L.), bergamot mint (*M*. *citrata* Ehrh.), and spearmint (*M*. *spicata* L.) EOs. The SM blend is encapsulated in a tiny soft beadlet for oral use. Peppermint essential oil (EO) is known for its ability to inhibit the growth of microorganisms, making it an effective natural alternative to synthetic antimicrobials [14,15,16,17,18,19,20,21]. Studies have shown that peppermint EO can be effective against antibiotic-resistant microorganisms, which is particularly important given the increasing prevalence of antibiotic resistance [22,23,24]. The active ingredient in mint EO, menthol, has strong antimicrobial properties against various microorganisms [14,16,17,25,26]. The antimicrobial effects of mint EO have been observed in a variety of applications. For example, mint oil has been used in oral hygiene products (toothpaste, mouthwash, and dental floss) due to its ability to kill halitosis- and gum disease-causing bacteria [27]. It has also been used in food preservation, as it can help to inhibit the growth of spoilage-causing microorganisms and extend the shelf life of food products [28]. Other potential applications of mint oil include its use in wound healing and skin care products. Mint oil has been found to have anti-inflammatory [29] and wound-healing properties [30,31]. Additionally, mint oil has been shown to have insecticidal properties [32,33], making it a potential natural alternative to synthetic insecticides.

Similar to peppermint oil, there is a plethora of research on the antimicrobial effects of Japanese mint [34,35], bergamot mint [36], and spearmint [16,18,19,20,37] EOs. Peppermint and spearmint EOs have been reported to have cleansing activity that may help protect the teeth [14,15,16,17,18,19,20]. While more research is needed to fully understand the antimicrobial effects of mint oils and their potential applications, the existing evidence suggests that they have excellent potential as a natural and effective way to combat harmful microorganisms. To the best of our knowledge, this work is the first study on healthy oral microbiomes in Egypt. A major part of the novelty of this study is identifying the oral microbiome in healthy volunteers before and after the consumption of natural antimicrobials. Previous studies on the oral microbiome determined by 16S rRNA gene sequencing in the Egyptian population included Hepatitis C virus (HCV) patients [38], liver disease [39], and dizygotic and monozygotic twins [40]. In this baseline pilot study, our objective was to evaluate the effects of SM beadlets on the oral microbiome profile in healthy Egyptian youth using the 16S rRNA gene sequencing technique.

## 2. Materials and Methods

### 2.1. Essential Oil Chemistry

SM is a proprietary blend (dōTERRA^®^, Pleasant Grove, UT, USA). The volatile constituents were analyzed by a Shimadzu GC–MS-QP2010 Ultra (Shimadzu Scientific Instruments, Columbia, MD, USA) with electron impact (EI) mode with 70 eV, using 40–400 *m*/*z* range scans with a scan rate of 3.0 scan/s, as previously described [41]. Compound identification was performed by comparing mass spectral fragmentation patterns (over 80% similarity match) and retention indices (RI) based on a series of homologous C8–C20 *n*-alkanes with those reported in databases [the NIST database and our in-house library] using the Lab Solutions GCMS post-run analysis software version 4.45 (Shimadzu Scientific Instruments, Columbia, MD, USA). A Gas Chromatography–Flame Ionization Detection (GC–FID) analysis was performed using a Shimadzu GC 2010 equipped with a flame ionization detector (Shimadzu Scientific Instruments, Columbia, MD, USA), as previously described [41], with a ZB-5 capillary column (Phenomenex, Torrance, CA, USA).

### 2.2. Ethical Review and Informed Consent

The Institutional Ethics Committee of the Faculty of Pharmacy, Suez Canal University, approved the study (IRB 2022207R1) in accordance with the 1964 Helsinki Declaration and its later amendments and comparable ethical standards. Written informed consent was obtained from all participants.

### 2.3. Study Population

For this investigation, a total of 20 healthy participants (out of 25 volunteers), including 10 males and 10 females, were recruited from the Faculty of Science (Suez University) and Faculty of Pharmacy (Suez Canal University) from January 2023 to February 2023. All participants read and signed a medical and nutritional history questionnaire along with an informed consent form. The inclusion criteria were age, 18–35 years; good general health (free of systemic diseases); good oral health (free of oral pathologies); available for the duration of the study; and signing the informed consent form [42,43,44]. The exclusion criteria were age under 18 years old or above 35 years old; heart diseases or blood pressure alteration that requires medication; renal, hepatic, or gastrointestinal disease that requires medication; diabetes; sexually transmitted diseases (STDs); HIV; HCV infection; antibiotic use for the last 3 months prior to the study; genetic disorders that could interfere with the evaluation of the study objectives; chronic obstructive pulmonary disease and asthma; any neoplastic lesion, cancer, or paraneoplastic syndrome; current radiotherapy or chemotherapy; pregnant or lactating women; smoking or vaping; more than 8 missing teeth, accounted for by third molar extractions, teeth extracted for orthodontic purposes, teeth extracted because of trauma, or congenitally missing teeth; orthodontic appliances; tumors or significant pathology of the soft or hard tissue of the oral cavity (such as LPO, erythroplakia, leukoplakia, and candidiasis); chronic dry mouth, as assessed through questioning by an experienced clinician; clinically meaningful halitosis as determined by organoleptic assessment by an experienced clinician; diagnosis of periodontitis; untreated carious lesions or oral abscesses; and use of alcohol-containing mouthwash [42,43,44]. All data remain anonymous, and no names were associated with any data resulting from this study. 

### 2.4. Treatment

A baseline investigation of the oral microbiome of the selected participants was performed by collecting saliva and swab samples before treatment. Four SM beadlets were chewed twice a day for 7 days. Post-administration saliva and swab samples were collected at the end of treatment.

### 2.5. Sampling

The sampling was performed at various locations in the oral cavity, including saliva, tooth surface, tongue, subgingival plaque around teeth, and buccal/palatal mucosa. For saliva collection, the volunteers were given 50 mL sterile collection tubes and were asked to allow 5 mL of saliva to flow naturally into the tube. An equal amount of sterile PBS was added to the saliva and then centrifuged at 1500 rpm for 10 min. The supernatant was then transferred to a new tube and centrifuged at 14,000 rpm for 20 min to obtain the bacterial pellet. All specimens were processed within 1 h from collection. To sample the soft tissue sites, swabs were used to collect samples from the tongue and buccal/palatal mucosa. Finally, sterile curettes were used for the hard tissue sites to collect supra- and subgingival plaque around the teeth.

### 2.6. Oral DNA Extraction

Genomic DNA of the oral microbiome was isolated from a combination of saliva (bacterial pellet) and soft and hard tissue samples using the Quick—DNA Fungal/Bacterial Miniprep Kit (Zymo Research, Irvine, CA, USA). Curettes and swabs were rinsed in the recommended volume of BashingBead™ Buffer with a 100 µL DNA shield (Zymo Research, Irvine, CA, USA). Then, pelleted cells (saliva) were resuspended in the same buffer. Proteinase K was added, and the cells were incubated at 65 °C for 1 h to increase cell lysis. After that, the DNA extraction was carried out according to the manufacturer’s protocol. Extracted DNA was visualized by gel electrophoresis; the DNA concentration and purity were quantified using a Nanodrop One spectrophotometer (Thermo Scientific, Waltham, MA, USA) and then stored at −20 °C. 

### 2.7. Amplicon Library Preparation and Sequencing

Genomic DNA extracted from each person (20–50 ng/μL) was used as a template for the amplification of the V3 and V4 regions of 16S rRNA. Briefly, amplicons were generated using Platinum^®^ PCR SuperMix (Thermo Scientific, USA) with indexed primers: Forward Primer with Illumina adaptor (underlined) 5′TCGTCGGCAGCGTCAGATGTGTATAAGAGACAGCCTACGGGNGGCWGCAG and Reverse Primer GTCTCGTGGGCTCGGAGATGTGTATAAGAGACAGGACTACH VGGGTATCTAATCC [45]. Thermal cycling consisted of denaturation at 95 °C (3 min), followed by 30 cycles of denaturation at 95 °C (30 s), primer annealing at 60 °C (30 s), and primer extension at 72 °C (45 s), followed by extension at 72 °C for 5 min. Amplicons were analyzed by gel electrophoresis to verify size (~400 bp, including adaptor sequences). Library preparation and sequencing were performed using the Illumina MiSeq platform (Illumina, San Diego, CA, USA) and accomplished at IGA Technology Services (Udine, Italy).

### 2.8. Bioinformatic Analyses and Ecological Statistics

Paired-end Illumina raw sequences were imported into Quantitative Insights in Microbial Ecology 2 (QIIME2 version 2023.2, Northern Arizona University, Flagstaff, AZ, USA) [46]. Positional quality plots were then used to decide the best positions for trimming and truncation. This was achieved using the QIIME2 command “qiime demux summarize”. Then, DADA2 [47] was used through QIIME2 with the qiime dada2 plugin to filter out noisy sequences (Northern Arizona University, Flagstaff, AZ, USA), thus correcting errors in marginal sequences, removing chimeric sequences, removing singletons, joining denoised paired-end reads, and then dereplicating these sequences, producing a unique amplicon sequence variant (ASV) feature table. The following parameters were used for the DADA2 pipeline: forward read sequences were truncated at 280, while reverse read sequences were truncated at 220. The first 24 bases were trimmed at 5′ end of each sequence for both forward and reverse sequences [48]. Taxonomy assignment of ASVs was performed by training an RDP naive Bayesian classifier at 99% sequence similarity against Greengenes 13_8 [49]. Further taxonomic classification of unrecognized features in our data set was performed using NCBI-BLAST. Quality-filtered reads from all time points were submitted to the NCBI Sequence Read Archive (SRA) under BioProject PRJNA1090251. Alpha diversity rarefaction values for each sample were generated in QIIME2 using Faith’s Phylogenetic Diversity metric using the “qiime diversity alpha rarefaction” command [50]. The overall taxonomic diversity before and after treatment was estimated using different alpha diversity metrics based on species richness that estimate the number of OTUs and Shannon diversity index. Beta diversity analysis was carried out by QIIME2 to estimate both unweighted and weighted UniFrac, Bray–Curtis, and distance matrix [50]. Alpha diversity (within sample variation) and beta diversity (between-sample variation) analyses were performed by running the QIIME2 script “qiime diversity core-metrics-phylogenetic” command. Taxonomic assignment tables generated by QIIME2 were imported into R to visualize the Shannon alpha diversity index, Pielou’s evenness index, and weighted Unifrac beta diversity. The Vegan R package was used to calculate Shannon’s index, which was visualized using the ggplot2 and patchwork packages. Weighted Unifrac distance was also calculated and visualized in R 4.2.2 using the Phyloseq package.

## 3. Results

### 3.1. Chemical Composition

SM is a proprietary blend from dōTERRA. Ingredients of SM beadlets include *Mentha piperita* EO, *M. canadensis* EO, *M. citrata* EO, *M. spicata* EO, medium-chain triglycerides, Guar gum, agar, glycerin, sodium alginate, and xylitol. The essential oil blend had a refreshing, minty scent. The volatile contents of the beadlets were analyzed by GC-MS and GC-FID. The major volatile components were menthol (46.19%), menthone (14.19%), linalool (4.40%), linalyl acetate (4.15%), menthyl acetate (3.87%), isomenthone (3.51%), carvone (3.31%), limonene (2.95%), 1,8-cineole (2.84%), neomenthol (2.37%), and β-caryophyllene (1.99%).

### 3.2. Demographic Features of the Study Population

Out of 25 initial volunteers and following the inclusion and exclusion criteria, 20 healthy participants (10 males and 10 females) with a mean age corresponding to 22.9 years (range 22–33) were enrolled in the study. The demographic features of the enrolled participants are summarized in Table 1. Overall, the mean number of teeth was 30.55 (range, 30–32). Twenty participants used manual toothbrushes, while only four used dental floss.

### 3.3. Microbiome Diversity and Composition

The oral microbiome samples were assessed by the high-throughput amplicon sequencing of 16S rRNA gene fragments. Six different specimens were collected from each subject, including saliva; three soft tissues sampled by sterile swabs (tongue and buccal/palatal mucosa); and two hard tissues sampled by sterile curettes (supra- and sub-gingival plaque). Genomic DNA extracted from each person (20–50 ng/μL) was used as a template for the amplification of the V3 and V4 regions of 16S rRNA using indexed primers. Following DNA amplification, samples were analyzed using the Illumina MiSeq platform by IGA Technology Services (Udine, Italy) for their community composition.

#### 3.3.1. Sequencing Data Profiles

After the quality trimming of the Illumina MiSeq raw sequencing data of the 40 oral samples, 1,482,526 read counts were maintained. The average read counts per sample were 37,490, while the minimum and maximum counts per sample were 11,536 and 63,725, respectively. The total number of ASV features that remained was 3026. The plateau was reached at 11,500 within the rarefaction curves. This sequencing depth largely captured the oral microbiomes; the max depth was 63,000 (Figure S1).

#### 3.3.2. Taxonomical Classification

**Figure 1:**
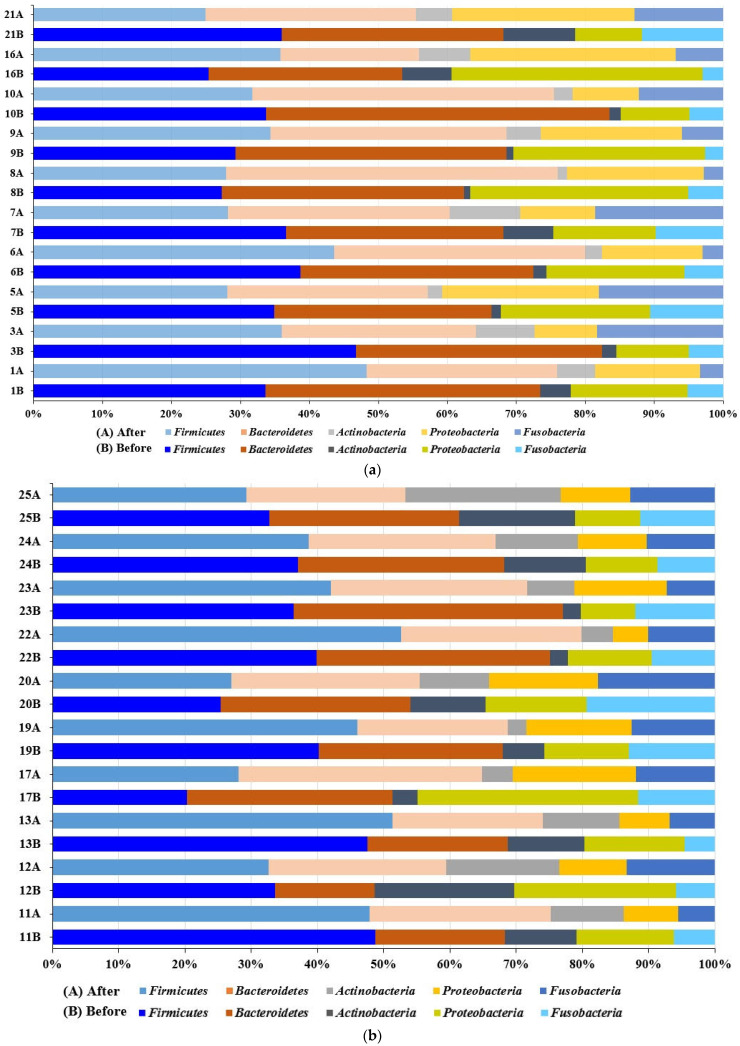
Relative abundance and distribution of the highest microbial phyla detected in the oral cavity. (**a**) Percentage distribution of detected phyla in males. (**b**) Percentage distribution of detected phyla in females. Participant IDs with B and A are before and after treatment, respectively.

Overall, the community compositions before and after treatment by the high-throughput sequencing of 16S rRNA are shown in Figure S2. The oral microbiome contains 60 families, 82 microbial genera, and 209 species. Firmicutes, Bacteroidetes, and Proteobacteria were the most abundant phyla in all study groups, followed by Fusobacteria and Actinobacteria. The phylum Firmicutes represents 20–50% of the total microbiome, followed by Bacteroidetes, which represents 19–47%, then Proteobacteria, Fusobacteria, and Actinobacteria, as shown in Figure 1.

**Figure 2:**
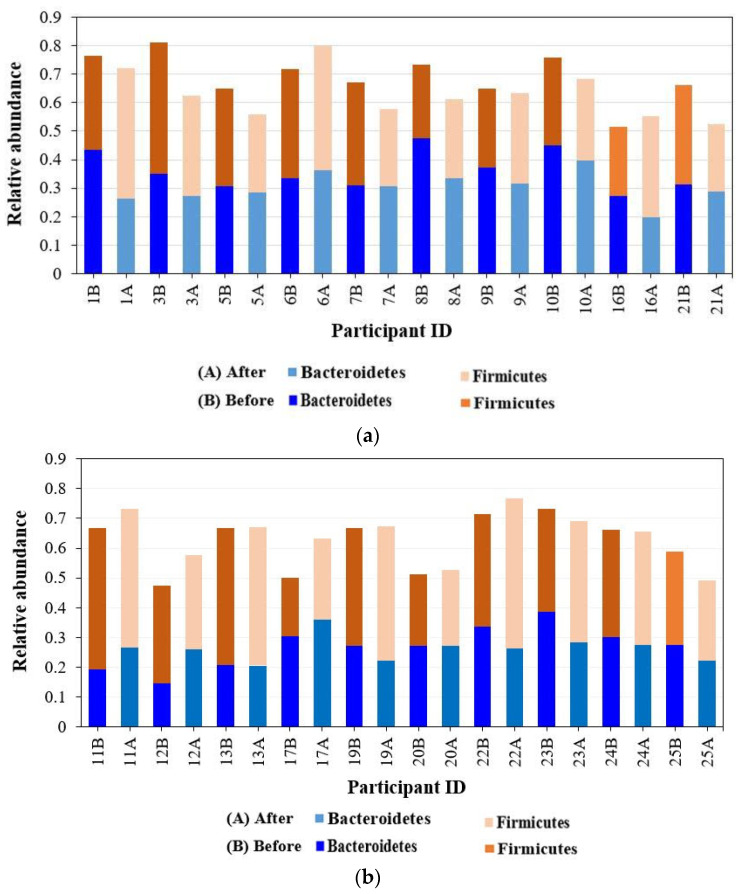
Relative abundance of the two taxa Firmicutes and Bacteroidetes detected in the oral cavity before and after mint usage. (**a**) Male. (**b**) Female. Participant IDs with B and A are before and after treatment, respectively.

It was observed that Firmicutes and Bacteroidetes made up to 80% of the community in some samples before SM usage, and the abundance of Bacteroidetes decreased or remained constant after treatment in the two groups. Firmicutes increased or remained constant after treatment in females, whereas Firmicutes decreased in males or remained constant (Figure 2).

**Figure 3:**
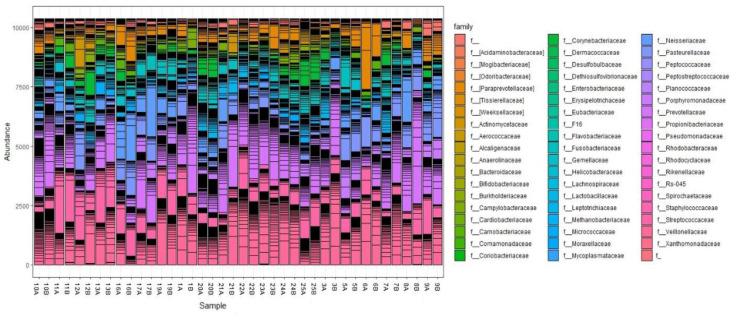
Barplot representation of families detected by 16S rRNA analysis in the clustered before and after treatment. Participant IDs with B and A are before and after treatment, respectively.

Overall, Archaea made up only 0.003%, while Gram-positive and Gram-negative bacteria represented 43.4% and 56.5% of the total sequences, respectively. The well-represented families in the microbiomes included *Veillonellaceae*, *Prevotellaceae*, *Streptococcaceae*, *Pasteurellaceae*, *Neisseriaceae*, *Fusobacteriaceae*, *Actinomycetaceae*, and *Paraprevotellaceae*. The relative abundance of the eight most abundant families (except *Veillonellaceae* and *Prevotellaceae*) varied significantly among participants. Notably, the highly abundant families *Prevotellaceae*, *Streptococcaceae*, *Pasteurellaceae*, *Actinomycetaceae*, and *Neisseriaceae* decreased after treatment. One notable finding was a decrease in or disappearance of *Spirochaetaceae*, a well-known cause of bad breath, after SM treatment, as shown in Figure 3. Moreover, the family *Odoribacteraceae* was represented by the infamous *Odoribacter* genus in one of the male samples. Interestingly, it decreased after SM use (Figure 3). Cyanobacteria, *Xanthomonadaceae*, and *Bifidobacteriaceae* were detected in females only. 

**Figure 4:**
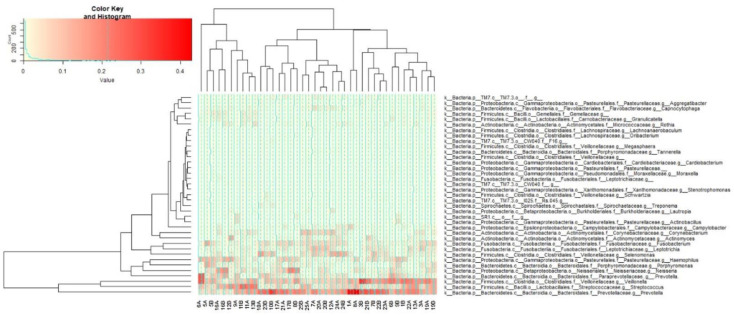
Heatmap representation of genera (with relative abundance of more than 1%) detected by 16S rRNA community analysis in each sample from each enrolled subject before and after treatment. Participant IDs with B and A are before and after treatment, respectively.

Figure 4 summarizes the relative abundance and distribution of the microbial genera detected in the oral cavity. Comparing the prevalence of the most abundant bacterial genera in males and females before and after mint usage (37 genera representing more than 1% of total bacteria) showed a clear difference in microbial distribution among males and females before and after treatment (Figure 4).

**Figure 5:**
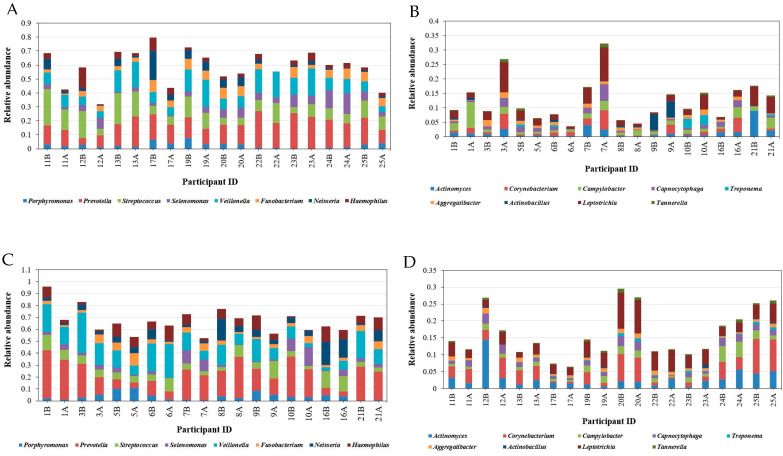
Relative abundance of taxa responsible for carries, halitosis, and periodontal disease across samples. (**A**) At genus level in males before and after treatment for highly abundant genera, (**B**) at genus level in males before and after treatment for moderately abundant genera, (**C**) at genus level in females before and after treatment for highly abundant genera, and (**D**) at genus level in males before and after treatment for moderately abundant genera.

In particular, *Prevotella* was the most abundant genus in the oral cavity (6 to 40% in males and 5 to 28% in females). It was followed by *Veillonella* and *Streptococcus*, which ranged from 4 to 33% and 3 to 26%, respectively. *Fusobacterium*, *Neisseria*, and *Haemophilus* were highly prevalent genera, ranging from 2 to 15% of the total bacteria detected. As expected, almost all the highly abundant genera decreased in about 70% of the samples in both males and females after treatment, including *Porphyromonas*, *Prevotella*, *Streptococcus*, *Neisseria*, and *Haemophilus.* Moreover, moderately abundant genera were decreased by SM, including Actinomyces, *Porphyromonas Lautropia*, *Actinobacillus*, *Aggregatibacter*, *Treponema*, *Leptotrichia*, *Campylobacter*, and *Capnocytophaga*, as shown in Figure 5. The patterns were not consistent in some genera; for example, *Veillonella* decreased in 80% of males but increased in 50% of females and decreased in the other females. It was also observed that Fusobacterium increased in 60% of male samples while decreasing in 90% of females. 

At the species level, *Prevotella melaninogenica* was the most prevalent (10.5% of the total detected species), followed by *Veillonella dispar*, representing 7.5% of the total detected species. *Haemophilus parainfluenzae* was the most abundant species belonging to the *Haemophilus* genus, with a relative abundance of 5%. *Neisseria subflava* was abundant, representing about 3.5% of the total species detected. However, *Treponema*, which causes bad breath, decreased from 0.06 to 3.28% (Figure 5).

#### 3.3.3. Alpha Diversity

**Figure 6:**
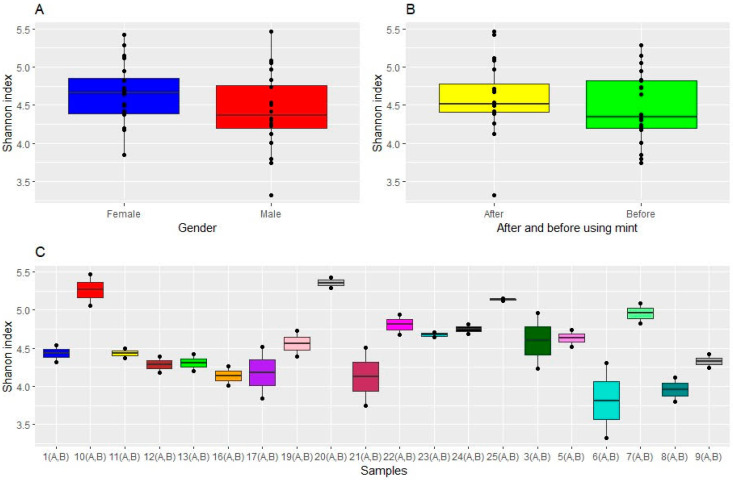
Alpha diversity indices of oral microbial communities across samples. Alpha diversity, as calculated by the Shannon alpha diversity index, differed (**A**) by gender, (**B**) before and after using mint, and (**C**) across samples. The only significant differences were between different participants.

**Figure 7:**
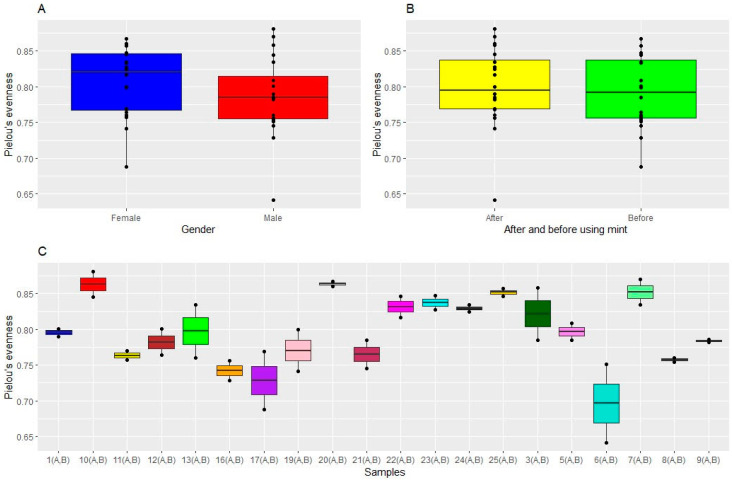
Alpha diversity indices of oral microbial communities across samples. Alpha diversity, as calculated by Pielou’s evenness index, differed (**A**) by gender, (**B**) before and after using mint, and (**C**) across samples. The only significant differences were between different participants.

The analysis of microbiome alpha diversity values in the twenty study participants, performed through the measurement of the Shannon index, evidenced, as expected, an appreciable inter-individual difference between participants, with alpha values ranging from 3.5 to 5.5. Moreover, statistically significant differences were observed (*p* = 0.036), suggesting that the microbiome differed between participants. The females’ microbiomes were more diverse than those of the males. Conversely, Shannon indices showed no statistically significant difference between microbiomes associated with females and males before and after treatment (Figure 6). These results were confirmed by Pielou’s evenness index (Figure 7).

#### 3.3.4. Beta Diversity

**Figure 8:**
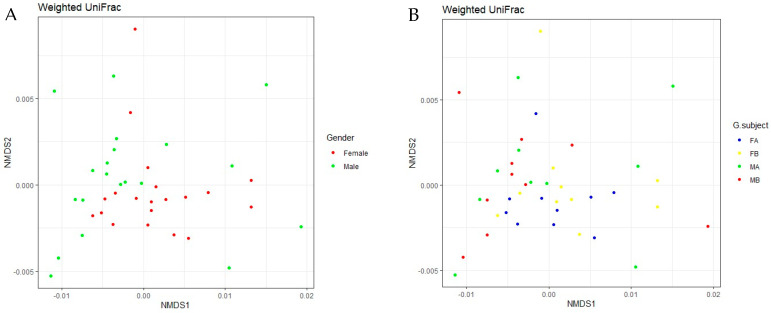
Beta diversity of oral microbiomes calculated with weighted UniFrac. (**A**) Gender; (**B**) gender and before and after treatment. Beta diversity analysis showed samples primarily separated by gender. FA, FB, MA, and MB are female after treatment, female before treatment, male after treatment, and male before treatment, respectively.

The dissimilarities between oral microbial communities were detected by measuring beta diversity by calculating the Bray–Curtis and Jaccard indices and the weighted UniFrac test. Beta diversity analyses showed a division of samples by gender identity and SM usage, although the patterns were not consistent (Figure 8).

## 4. Discussion

The use of molecular 16S rRNA gene NGS techniques has enabled a more comprehensive understanding of the bacterial component of the oral microbiome compared with culture-based methods. These techniques have revealed differences between the male and female oral microbiomes (HOMs) and the effect of treatment on oral microbiomes. In this study, we analyzed the oral microbiome of individuals in a normal, healthy state in Ismailia and Suez, Egypt, by profiling oral microbiota using 16S rRNA next-generation sequencing.

The present study aimed to examine changes in the oral microbiome of healthy adult participants before and after treatment with mint beadlets without further dilution, using NGS as the analysis method. To achieve this goal, we systematically analyzed the oral microbiomes of 20 healthy participants (10 males and 10 females) using NGS for human oral microbiome characterization, both on site-specific oral samples (hard and soft tissues) and on saliva specimens. Our results showed that there were 3026 operational taxonomic units (OTUs) shared with the oral microbiota in each of the saliva and hard and soft tissues of the 20 individuals. We found five phyla in healthy individuals, including Proteobacteria, Firmicutes, Actinobacteria, Fusobacteria, and Bacteroidetes. These five phyla accounted for 90–98% of the entire oral microbiome. Thus, in agreement with other studies, the five phyla account for 80–95% of the entire oral microbiome [51,52].

In the study, it was discovered that healthy individuals had over 82 microbial genera in their HOMs. However, there were statistically significant differences between the enrolled participants. Interestingly, this study found no significant differences related to gender, which is consistent with previous research that also found no gender-related differences in oral microbiome diversity [51]. Additionally, no significant differences were observed when using hygiene devices (such as floss) or mint. However, this study did find that the female oral microbiome composition was more variable than that of the males after treatment, suggesting a diverse alpha diversity value in the male versus female group. At the genus level, this study found a total of 37 distinct genera present at a 1% abundance.

This study found that there were several abundant genera present in all participants, including *Prevotella*, *Streptococcus*, *Selenomonas*, *Veillonella*, *Fusobacterium*, *Neisseria*, *Haemophilus*, *Actinomyces*, and *Porphyromonas*, which were previously characterized. Additionally, *Corynebacterium*, *Campylobacter*, *Capnocytophaga*, *Treponema*, *Aggregatibacter*, *Actinobacillus*, *Leptotrichia*, *Tannerella*, and *TM7* were present in lower abundance. This suggests the existence of a core microbiome in the oral environment, similar to what has been proposed in other studies [51,53]. Furthermore, most oral taxa found in unrelated healthy individuals were similar, indicating the presence of a healthy microbiome [54,55,56]. *Prevotella melaninogenica* was found to be the most abundant species, and its abundance was higher in participants aged 25 and 32 compared with those aged 21, in line with previous studies on human microbiomes within different age groups [57]. Overall, this study identified over 200 bacterial species with high accuracy (>99%), including *Prevotella melaninogenica*, *Haemophilus influenzae*, *Veillonella dispar*, *Neisseria subflava*, and *Rothia mucilaginosa*.

Our study found that a subject’s gender and use of SM were the primary factors influencing differences between participants. Interestingly, the data revealed that each subject was significantly associated with a diverse microbial composition. We also sought to explore the impact of microorganisms that make up the human oral microbiome in healthy conditions. These findings suggest that the microbiome of the healthy subject (HOM) has common recognizable characteristics, whatever their gender, and that SM treatment did not change the microbiome diversity while affecting their abundance. It is worth noting that menthol, the major EO constituent in this study, has been shown to possess several biological properties, including antimicrobial, anticancer, and anti-inflammatory activities [18,19,20,21,25,26,29].

According to our results, SM treatment decreased the abundance of *Streptococcus*, *Veillonella*, *Actinomyces*, and *Leptotrichia*. These genera have been previously reported as abundant in severe early childhood caries subjects [58]. Additionally, several studies on halitosis and periodontal disease-causing bacteria have identified *Actinomyces* spp., *Bacteroides* spp., *Dialister* spp., *Eubacterium* spp., *Fusobacterium* spp., *Leptotrichia* spp., *Peptostreptococcus* spp., *Porphyromonas* spp., *Prevotella* spp., *Selenomonas* spp., *Solobacterium* spp., *Tannerella forsythia*, and *Veillonella* spp. as bacteria that are most related to halitosis and periodontal diseases [59,60,61]. Notably, all of these genera were affected and decreased after using mint. Moreover, *Streptococcus* spp., the most common pathogens involved in dental caries pathogenesis, also decreased in prevalence after mint usage. Our findings align with previous research indicating that menthol is effective against various bacteria, such as *S. aureus*, *S. mutans*, *S. faecalis*, *S. pyogenes*, and *L. acidophilus* [62,63]. The antimicrobial activity of mint oil against *Streptococcus mutans*, as well as the antibiofilm activity of the components of essential oils from *Mentha* spp., has been reported [64]. Furthermore, *Leptotrichia* spp., known to ferment carbohydrates and produce lactic acid that may be involved with tooth decay, decreased after treatment. *Rothia dentocariosa* and *R. aeria* were present in the oral cavity before mint usage, comprising 0.2% to 5% of the content, but decreased after mint usage to a range of 0.1% to 2.7%, approximately half of the initial content. While the *Gemella* genus increased after using mint, it was observed that *Gemella* species have clear ecological preferences in the oral cavity of healthy humans that maintain health in a state of equilibrium [65]. Our findings suggest that *Gemella* sp. increased while *Porphyromonas gingivalis* decreased, in alignment with previous studies. It was reported previously that *G. hemolysis* inhibits the growth of *P. gingivalis,* a species closely related to periodontal disease and halitosis, in vitro [66]. Interestingly, *Odoribacter*, known for producing high numbers of volatile organic compounds (VOCs) that cause unpleasant odors, decreased after using SM. Regarding potential harmful effects, essential oils should be used with care. Excessive use or undiluted application can cause irritation. Additionally, some essential oils may disrupt oral homeostasis by affecting the natural balance of oral microbiota. Therefore, moderation and proper dilution are crucial to avoiding adverse effects. The current study used a blend of mint oils diluted in coconut oil (medium-chain triglycerides), as described in Section 3.1.

Recently, oral corynebacteria have gained significant attention due to their crucial role in the oral microbiome’s biogeography [67]. According to further metagenomic sequencing analyses, *Corynebacterium* spp., particularly *C. matruchotii*, are among the most common species found in adults, suggesting that *Corynebacterium* sp. is part of the core oral commensals [68]. It is worth noting that *Corynebacterium* species are present in the conjunctiva of healthy adults and are non-pathogenic. Our results show an increase in *Corynebacterium* and a decrease in *Streptococcus sanguinis*. Commensal microbes, such as *Corynebacterium* spp., play crucial roles in maintaining oral health through mechanisms involving hydrogen peroxide production and membrane vesicle secretion, which can inhibit pathogenic species and modulate host immune responses [69].

## 5. Conclusions

The current study successfully evaluated the effects of SM beadlets on the human oral microbiome. The results showed a significantly decreased abundance of the *Prevotella*, *Streptococcus*, *Neisseria*, and *Haemophilus* microbial genera and families after treatment. Although some genera showed inconsistent patterns, it was observed that SM treatment effectively reduced the abundance of several bacteria associated with halitosis and periodontal disease, such as *Actinomyces* and *Streptococcus*. Moreover, *Corynebacterium* species increased after treatment. These findings strongly suggest that SM beadlets have the potential to improve breath odor. However, further research is needed to fully understand the antimicrobial effects of mint oils and their potential applications in maintaining good oral health.

## Figures and Tables

**Table 1:** Demographic features of the study population.

Participant ID	Gender	Age	No. of Teeth	Oral Hygiene Devices at Home	City of Residency
1	Male	23	32	None	Ismailia
3	Male	23	32	Floss	Cairo
5	Male	24	32	None	Cairo
6	Male	23	30	None	Ismailia
7	Male	23	32	None	Sinai
8	Male	22	32	None	Ismailia
9	Male	22	31	None	Ismailia
10	Male	23	31	None	Ismailia
11	Female	21	32	None	Eltour
12	Female	21	32	None	Suez
13	Female	21	32	None	Suez
16	Male	21	32	None	Suez
17	Female	21	32	None	Suez
19	Female	21	31	None	Suez
20	Female	21	32	None	Suez
21	Male	23	32	Floss	Ismailia
22	Female	25	32	None	Ismailia
23	Female	22	32	None	Ismailia
24	Female	25	31	Floss	Suez
25	Female	33	32	Floss	Ismailia

## Data Availability

The sequence data presented in this study are openly available in NCBI Sequence Read Archive (SRA) under BioProject PRJNA1090251. [NCBI] [https://www.ncbi.nlm.nih.gov/bioproject/] [PRJNA1090251].

## Supplementary Materials

The following supporting information can be downloaded at https://www.mdpi.com/article/10.3390/microorganisms12081538/s1, Figure S1: Alpha-rarefaction curve. Figure S2: Taxonomic composition of oral microbiome.
