# Supplementary material for: Effects of Mint Oils on the Human Oral Microbiome: A Pilot Study
Source: Microorganisms. 2024 Jul 27;12(8):1538. doi: 10.3390/microorganisms12081538 (PMC11356387; doi:10.3390/microorganisms12081538)
Supplement: Supplementary file 1 [file microorganisms-12-01538-s001.zip › microorganisms-3060009-supplementary.pdf]

microorganisms

Article

Effects of Mint Oils on the Human Oral Microbiome: A Pilot Study

Supplementary figures

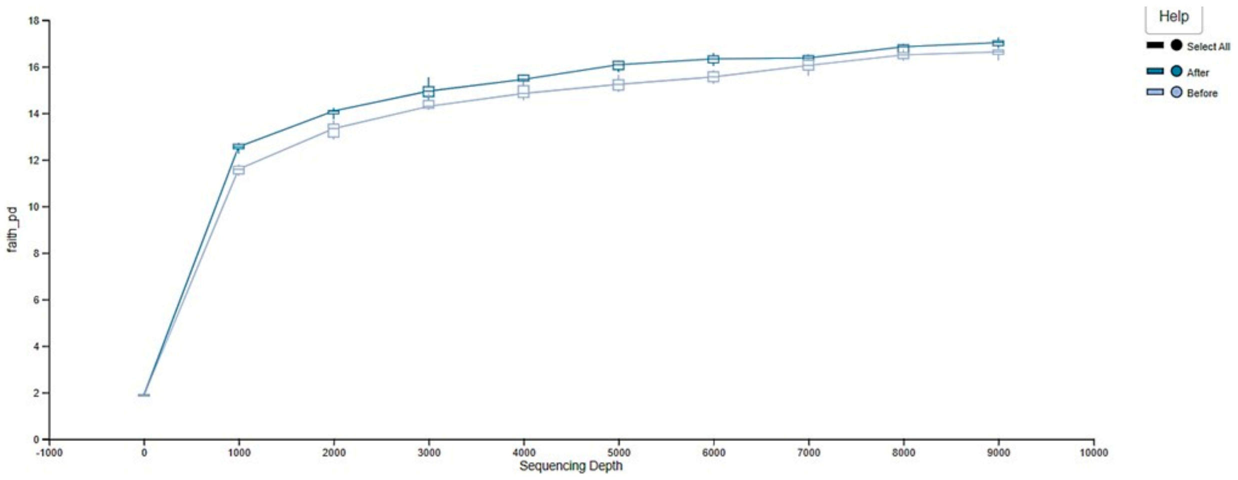

Figure S1. Alpha-rarefaction curve.

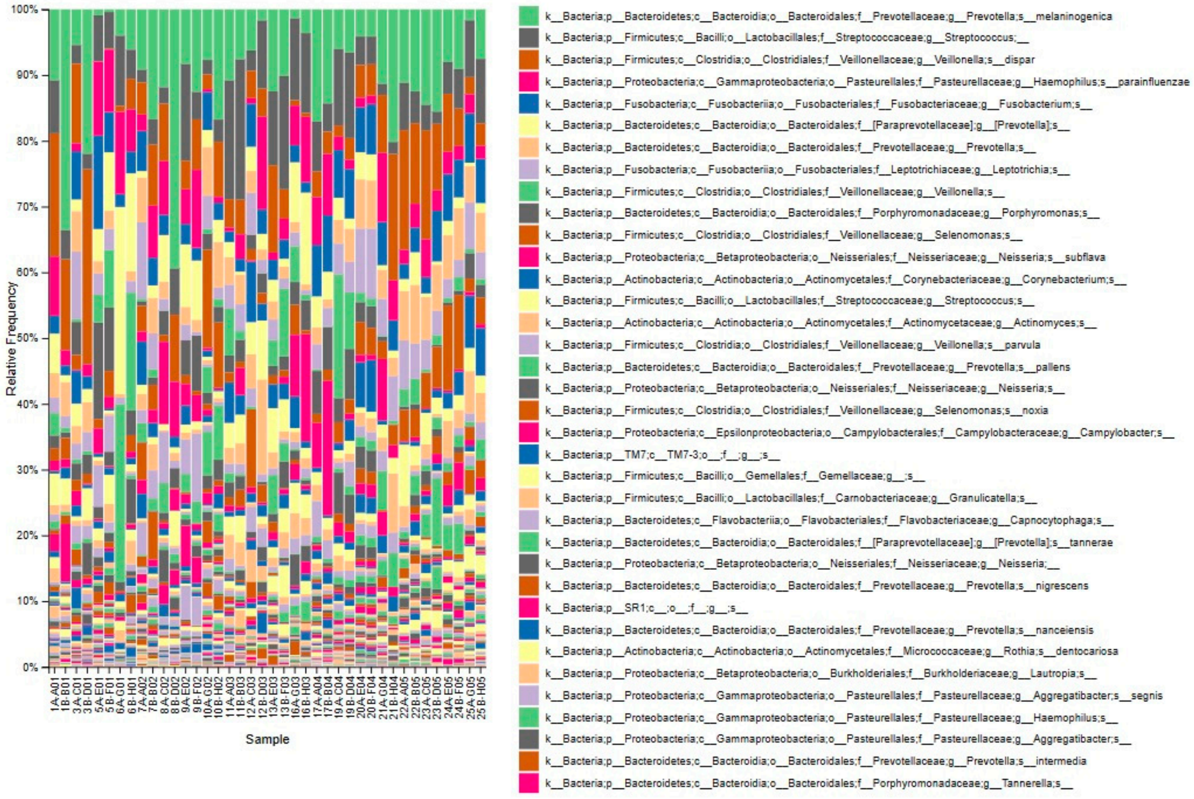

Figure S2. Taxonomic composition of oral microbiome.
